# Dipole-driven self-organization of zwitterionic molecules on alkali halide surfaces

**DOI:** 10.3762/bjnano.3.32

**Published:** 2012-03-27

**Authors:** Laurent Nony, Franck Bocquet, Franck Para, Frédéric Chérioux, Eric Duverger, Frank Palmino, Vincent Luzet, Christian Loppacher

**Affiliations:** 1 Aix-Marseille Univ, IM2NP, Faculté des Sciences de St. Jérome, F-13397 Marseille France; CNRS, IM2NP, Faculté des Sciences de St. Jérome, F-13397 Marseille France; 2 Institut FEMTO-ST, Université de Franche-Comté, CNRS, ENSMM, 32, Avenue de l’Observatoire, F-25044 Besancon Cedex, France

**Keywords:** alkali halide surface, noncontact atomic force microscopy, organic molecule, self-organization, zwitterion

## Abstract

We investigated the adsorption of 4-methoxy-4′-(3-sulfonatopropyl)stilbazolium (MSPS) on different ionic (001) crystal surfaces by means of noncontact atomic force microscopy. MSPS is a zwitterionic molecule with a strong electric dipole moment. When deposited onto the substrates at room temperature, MSPS diffuses to step edges and defect sites and forms disordered assemblies of molecules. Subsequent annealing induces two different processes: First, at high coverage, the molecules assemble into a well-organized quadratic lattice, which is perfectly aligned with the <110> directions of the substrate surface (i.e., rows of equal charges) and which produces a Moiré pattern due to coincidences with the substrate lattice constant. Second, at low coverage, we observe step edges decorated with MSPS molecules that run along the <110> direction. These polar steps most probably minimize the surface energy as they counterbalance the molecular dipole by presenting oppositely charged ions on the rearranged step edge.

## Introduction

The adsorption of organic molecules on a crystalline substrate surface is governed by a delicate balance between the molecule–molecule (MM) and the molecule–substrate (MS) interaction. The latter can strongly depend on the registry between the organic layer and the inorganic substrate, especially if coincidences between the two lattices are possible (for an overview of the different epitaxial ordering see, for example, [1–2]). On the one hand, for metallic and semiconducting substrate surfaces, there often exists quite a strong MS interaction, which can either be caused by covalent binding or by weak overlap between the π-orbitals of the organic molecule and the electronic states of the surface. On the other hand, for insulating substrates, the vertical MS interaction is often much weaker than, for example, the intermolecular π-stacking of the organic molecules. Although for certain molecules π-stacking can lead to the formation of one-dimensional wires [3], in general, for organic–inorganic heteroepitaxy (OIHE) on insulating substrates the growth mode is often governed by a dewetting process [4] as the MM interaction dominates.

In the last few years, many studies have been focused on alkali halide surfaces as model systems for the study of OIHE on insulating substrates (for an overview see, for example, [5]). These surfaces are nonreactive, easy to prepare by cleavage of single crystals or by vapour deposition of thin films on metal substrates, and they are atomically well-defined. Different routes have been proposed to circumvent the problem of dewetting since it is the control of a few, down to even single, homogeneous and well-ordered molecular layers that is desired for many applications in molecular (opto-)electronic devices.

As has been shown by Loppacher et al. [6], large and ordered structures are obtained if either the MM or the MS interaction dominates. In the former case, the structures mostly grow in three-dimensional crystallites [7–10]. Only for systems in which the MM interaction was directional, as for example by H-bonding [11] or by covalent bonding [12–13], layer-by-layer growth or even one-dimensional growth [14] was observed. When the MS interaction dominates, monolayer (ML) growth can be obtained more easily. For example, a few systems have been reported in which a metastable phase with a point-on-point epitaxy [15–16] or other well-defined epitaxies [17] were found and single molecular layers were observed. Furthermore, structured monolayer growth was obtained on a nanostructured surface [18].

In our work we study the influence of the molecular dipole on the adsorption of zwitterionic molecules on ionic-crystal (100) template surfaces. The crystals chosen (NaCl, KCl, RbCl, and KBr) all show the same structure (face-centered cubic, or rock salt) and thus provide an identical quadratic pattern of alternating electric charges on the surface, but with a different lattice constant (see below in Table 1). In other heteroepitaxial systems it was often observed that the orientation of an incompressible overlayer depended not only on the parameters during the sample preparation (substrate temperature, evaporation rate), but also on the lattice mismatch between the two structures [9,19]. Therefore, we have chosen the above-mentioned model substrates in order to verify whether the electrostatic MS interaction between the molecular charge distribution and the ions on the substrate surface could be used to force the molecular arrangement along equally charged 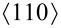 oriented substrate lines, regardless of the substrate lattice constant.

## Experimental

**Figure 1:**
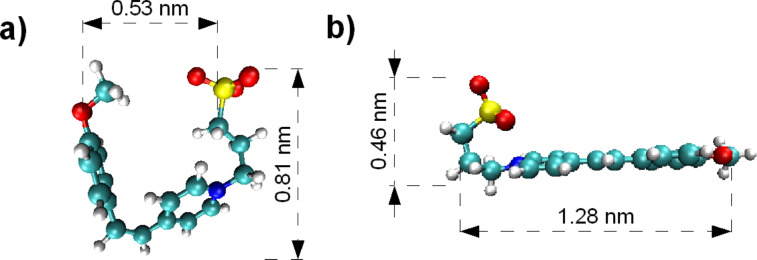
MSPS can have two conformations, namely the agraffe-like cis (a) and the scorpion-like trans (b) isomerization.

The molecule we used is the zwitterion 4-methoxy-4′-(3-sulfonatopropyl)stilbazolium (MSPS). MSPS molecules were synthesized in accordance with the method previously described by Serbutoviez et al. [20] and Makoudi et al. [21]. MSPS is composed of a sulfonato endgroup (SO_3_^−^), which carries a negative charge and which is linked via an alkyl-chain to a pyridinum ring carrying a positive charge (N^+^). MSPS molecules adopt two main conformations corresponding to the cis/trans isomerization of the C–C double bond. Cis (agraffe-like) and trans (scorpion-like) are described in Figure 1. However, only the scorpion-like isomer is obtained after the synthesis, because it is more stable than the cis isomer. The permanent electric dipole of the trans isomer is 16.8 D, the total length of this isomer is 1.28 nm. Due to the isomerization of the C–C double bond, the cis isomer is shorter than the trans isomer (0.53 vs 1.28 nm).

The ionic single-crystal substrates (MaTecK GmbH, Jülich, Germany) were cleaved ex situ and annealed in situ (UHV conditions) to 150–250 °C in order to obtain clean terraces and well-defined step edges.

The molecules were deposited from home-built pyrolytic boron nitride crucibles with the substrate kept at room temperature. The deposition rate was monitored by a quartz micro balance and set to approximately 0.5 ML/min. Large-scale ordering of the deposited molecular layers could only be achieved after subsequent annealing of the substrate to ≈110 °C for 15–30 min. Annealing to lower temperatures only affected the substrate surface a little; choosing higher temperatures resulted in desorption of the molecules.

Noncontact atomic force microscopy (NC-AFM) measurements were performed in situ under UHV conditions (<2·10^−10^ mbar) by means of a variable temperature AFM (VT-AFM, Omicron Nano Technology GmbH, Taunusstein, Germany) equipped with RHK electronics (SPM1000, RHK Technology, Troy, MI 48083, USA). Cantilevers used are PPP-NCL (Nanosensors, Neuchâtel, Switzerland) with resonance frequencies of ≈150 kHz, spring constants of ≈50 N/m, and quality factors of ≈35,000. Typical oscillation amplitudes were 5–10 nm (10–20 nm peak-to-peak). The cantilevers were heated in situ to ≈150 °C for one hour in order to remove contaminants from the tip. In NC-AFM, the oscillation amplitude of the cantilever is kept constant by an oscillation feedback controller and the topography is regulated by keeping the frequency shift Δ*f* constant. The contact potential difference between the tip and the sample was compensated by applying the corresponding bias voltage to the tip (static, no feedback). For image evaluation we used the WSxM software [22].

## Results and Discussion

**Figure 2:**
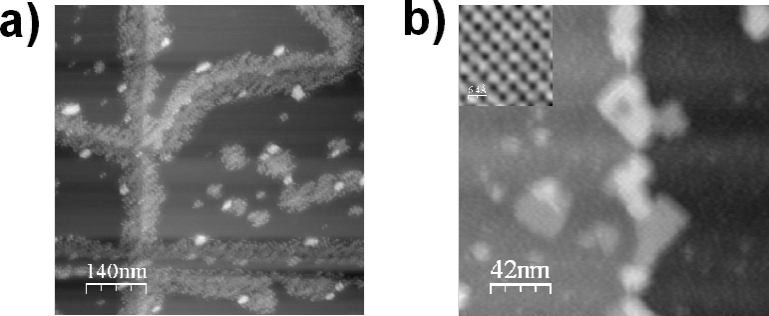
0.2 ML of MSPS evaporated onto KCl. (a) displays the NC-AFM topography after deposition at RT (Δ*f* = −59 Hz, *A*_0_ = 7 nm), (b) shows the surface after annealing to 110 °C for 15 min (Δ*f* = −40 Hz, *A*_0_ = 7 nm). The substrate orientation is shown in the inset.

**Figure 3:**
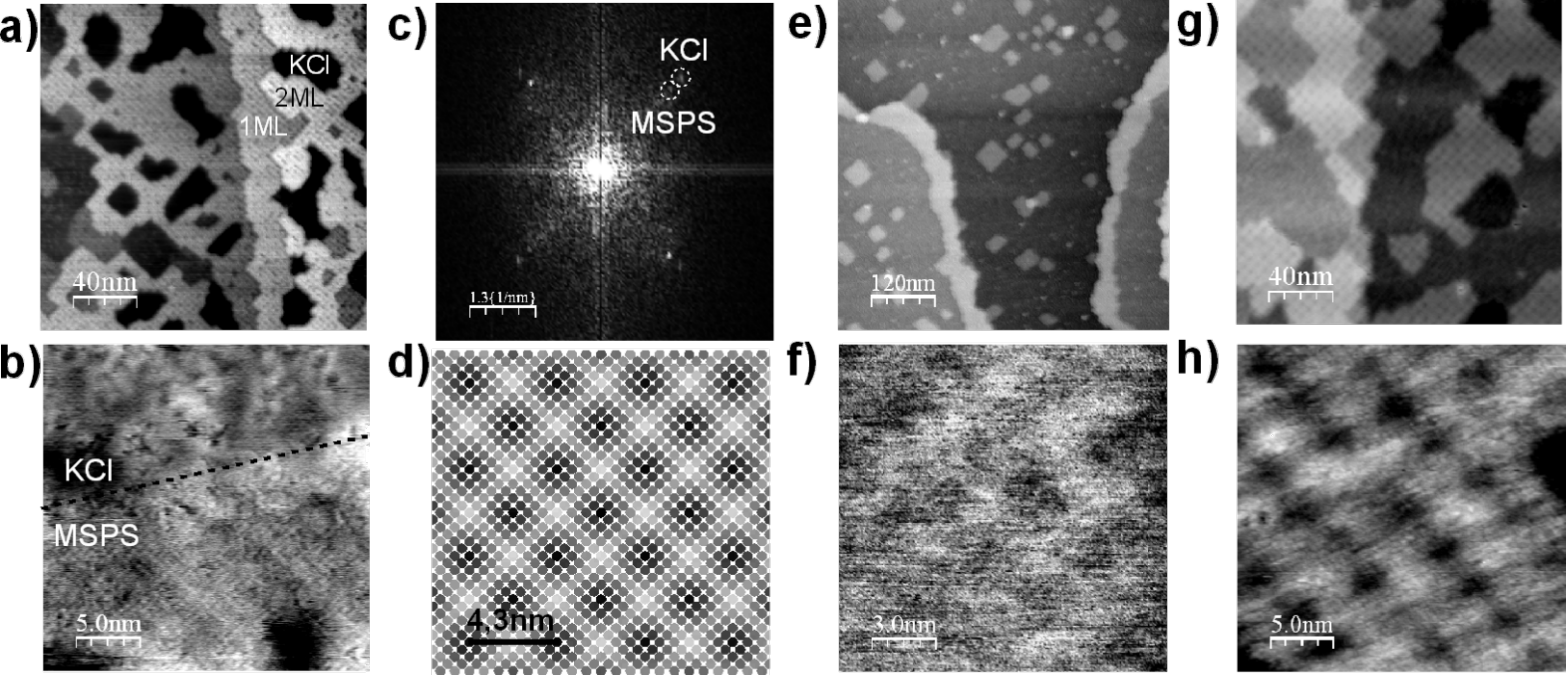
(a) Topography image of ≈1 ML of MSPS adsorbed on KCl (Δ*f* = −75 Hz, *A*_0_ = 7 nm); (b) shows a close up view on an MSPS island boundary with both, the MSPS and the KCl substrate imaged with molecular resolution (Δ*f* = −17 Hz, *A*_0_ = 5 nm). (c) Fourier transform of the image displayed in (b), two quadratic and parallel lattices are observed; MSPS has a slightly larger lattice constant. (d) simulation of a Moiré pattern obtained by superimposing the lattices of KCl and MSPS. (e) Topography image of ≈0.3 ML MSPS on RbCl (Δ*f* = −60 Hz, *A*_0_ = 7 nm) with a close up view of an MSPS island in (f) (Δ*f* = −230 Hz, *A*_0_ = 7 nm). (g) Topography image of ≈0.5 ML MSPS on KBr (Δ*f* = −25 Hz, *A*_0_ = 10 nm) with a close up view on an MSPS island in (f) (Δ*f* = −30 Hz, *A*_0_ = 10 nm).

The topography image of ≈0.2 ML MSPS deposited on a clean KCl substrate surface is depicted in Figure 2a. MSPS diffuses to step edges and impurities and forms disordered amorphous islands, but with a more or less uniform height, and no formation of larger clusters or double layers. Most probably, the strong electrostatic MS interaction hinders both a three-dimensional growth and a reorganization into closely packed ordered islands on a large scale. It is only after a subsequent annealing cycle (110 °C for 15 min) to temperatures close to the sublimation temperature of MSPS (≈120 °C) that large-scale ordering of MSPS into rectangular islands is observed (Figure 2b). These islands are all oriented along the 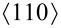 direction of the substrate (see inset of Figure 2b for substrate orientation) and show a regular, quadratic Moiré pattern, several nanometers large, parallel to the island boundaries. With the annealing cycle, we also observe a rearrangement of the KCl substrate surface, which will be discussed at the end of this section. For the moment we would just like to mention that the diffusion of MSPS, with its strong electric dipole, on an ionic surface can create atomic-scale defects (see for example the upper part of Figure 3b).

For a detailed investigation of the lattice parameters of both the molecular protrusions in the MSPS islands and the Moiré pattern respectively, we proceeded as follows: first, atomic-resolution images of the substrate surface (e.g., inset of Figure 2b) were used to determine the substrate orientation and to calibrate the scanner; second, the MSPS islands (and, if possible, simultaneously the substrate surface) were imaged on the molecular length scale; and third, large-scale images of the islands with the Moiré pattern were acquired. All images were drift corrected and evaluated in order to give the most accurate values for the experimentally determined lattice constant of MSPS *c*_msps,exp_ as well as for the Moiré pattern *l*_Moiré,exp_.

Figure 3a shows the topography image of ≈1 ML of MSPS on KCl. The image was taken after the annealing cycle, which induced the self-organization of the molecules. A large-scale ordering into islands one and two ML thick with a regular quadratic Moiré pattern of *l*_Moiré,exp_ = 30 ± 2 Å is observed. On a molecular length scale, quadratic lattices are measured for both the molecular protrusions as well as for the substrate (Figure 3b). A Fourier-transform of the image in Figure 3b reveals two equally oriented quadratic lattices with the MSPS lattice measuring *c*_msps,exp_ = 5.1 ± 0.1 Å (Figure 3c).

As mentioned above, MSPS has several conformational degrees of freedom and thus it is difficult to determine its exact conformation in the well-ordered islands observed on KCl. Makoudi et al. [23] used scanning tunneling microscopy (STM) to measure MSPS on Au(23 23 21) and observed a parallelogram unit cell with dimensions of 1.1 × 0.5 nm^2^, and the molecules were adsorbed in the so-called scorpion-like conformation. The dipole moment of the molecule, which in this conformation is oriented perpendicular to the substrate surface, could only be imaged with low contrast, or not at all, by STM, due to its reduced conductivity and the fact that it is flexible.

**Figure 4:**
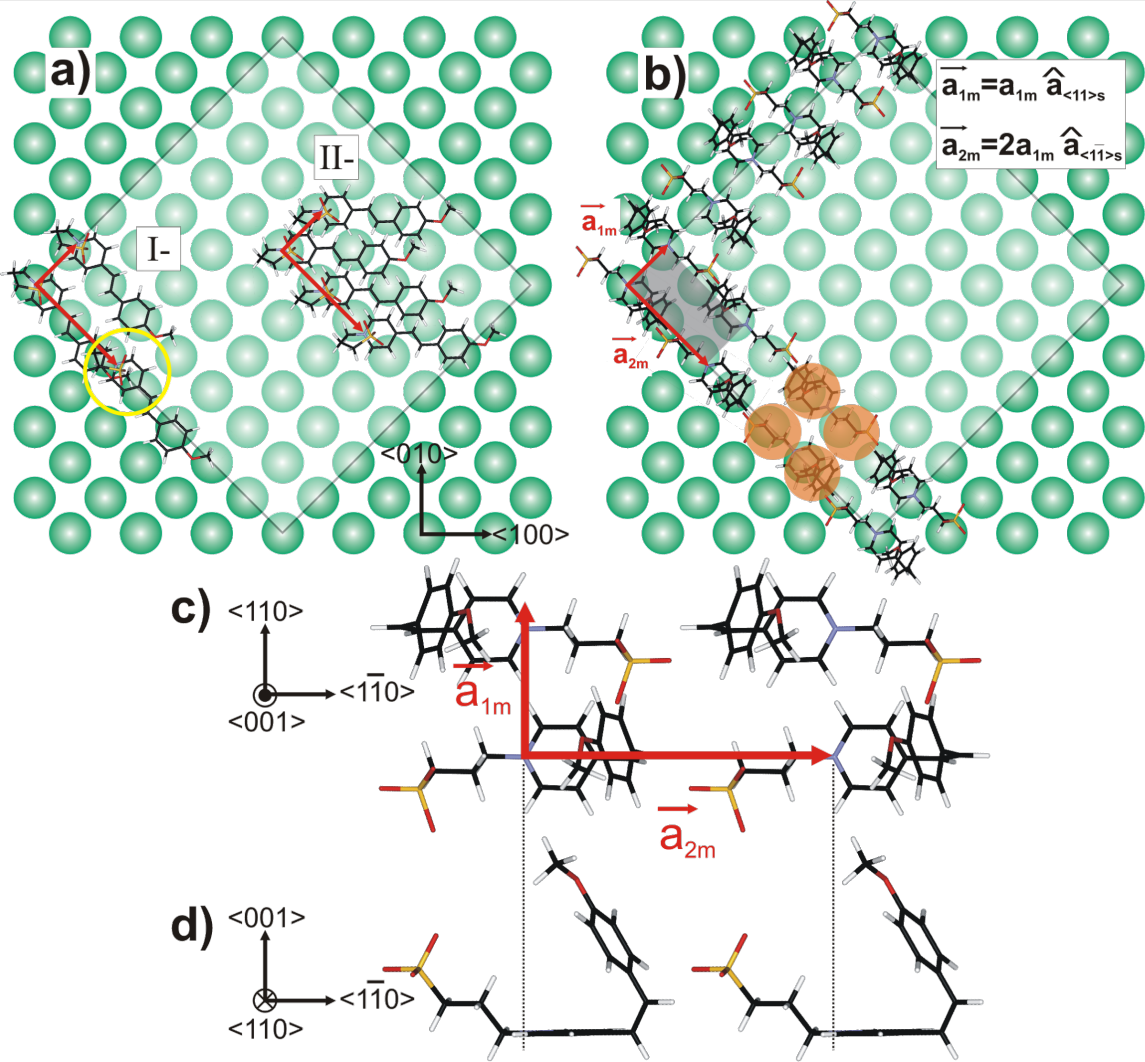
Conformations of MSPS on KCl(001). Only the positions of the substrate anions have been drawn. In (a) and (b), the white area depicts one patch of the molecular Moiré, as experimentally derived on KCl(001): 
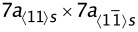
 = 31 × 31 Å^2^, 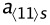 = 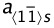
= 4.65 Å being the interionic distances along the 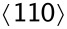 and 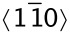 directions, respectively. (a) MSPS adsorbed in the scorpion-like conformation with molecular rows parallel to the 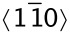 direction (I) and to the 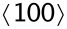 direction (II). Arrangements (I) and (II) do not match the experimental findings (see text). (b) MSPS in the agraffe-like conformation with the corresponding rectangular unit cell: *a*_1_*_m_* × *a*_2_*_m_* = 5.1 × 10.2 Å^2^ aligned along the 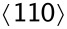 and 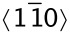 directions, respectively. Note that *a*_2_*_m_* is twice as large, which reflects the fact that each molecule is composed of two protrusions along this molecular axis (orange areas). In the agraffe-like conformation, the MM interaction is increased due to the interaction between the zwitterionic part of one molecule and the dipole moment of the anisyl part of the neighboring molecule, as illustrated in the top view (c) and in the side view (d).

In order to draw conclusions about the most probable conformation of the molecule adsorbed on the surface, we compare the experimentally determined parameters with the possible molecular conformations (the so-called scorpion- or agraffe-like conformation obtained by numerical simulation of a single molecule in vacuum, see Figure 1). Note that the comparison between the apparent size of the quadratic molecular unit cell (5.1 × 5.1 Å^2^) and the distance between the ends of the molecule in the two former conformations (1.28 or 0.53 nm, respectively) requires each molecule to be imaged as (at least) two protrusions to account for the experimental images. To illustrate this issue, we have sketched the two adsorption conformations of the molecule on KCl(001) in Figure 4. In the case of the agraffe-like conformation, one might be tempted to consider a single protrusion (0.53 vs 0.51 nm) per molecule. However, in this situation, it is not possible to account for the experimental molecular unit cell as there would be a huge steric hindrance due to the molecular aspect ratio. From the fact that the molecular islands grow in a twofold symmetry, we conclude that one molecule must be represented by exactly two protrusions.

For the scorpion-like conformation, similar to the STM experiments [23], the two protrusions observed in the experimental pattern must be the aromatic rings, which are separated by 6.5 Å. This distance is too large to fit the experimentally observed pattern when the molecules are aligned along the 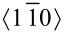 direction of the substrate (Figure 4a, I). When aligned along the 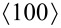 direction (Figure 4a, II), there is steric hinderance between the aromatic rings and a molecular film can only be formed when the aromatic rings are tilted by ≈40 °C with respect to the substrate surface. To our knowledge, such a film would only organize on a large scale if there was a significant MM interaction, as is, for example, the case for molecules with strong H-bonding [11]. It is therefore very unlikely that the molecules adopt the scorpion-like conformation, as first the molecular distances do not fit the experimental ones, and second, the formation of a homogeneous layer would not be promoted by MM interaction.

In contrast, for the agraffe-like conformation, the distance between the two ends of the molecule (i.e., 5.3 Å as depicted in Figure 1) is close to the experimentally observed value of 5.1 Å. The molecule adsorbs with its pyridine ring parallel to the surface and the N^+^ charge on a line of substrate anions (see Figure 4b). Furthermore, if the molecules are rotated alternately by 180°, along the 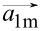 direction, a MM interaction can be established in both directions of the molecular unit cell. This interaction is formed between the zwitterionic part of one molecule and the anisyl part (i.e., methoxyphenyl) of the neighboring molecules (Figure 4c and Figure 4d). An additional indication in favor of the agraffe-like conformation is the fact that the highly ordered organic layers are only formed after subsequent annealing cycles up to 110 °C. This temperature is sufficiently high to induce the isomerization of MSPS on the surface, as the isomerization energy for stilbene (i.e., a molecule that represents the central part of MSPS only) is estimated to be ≈0.2 eV [24]. We therefore think that it is the agraffe-like conformation that the molecules adopt in our experiment and that the molecular unit cell must be rectangular, measuring 5.1 Å × 10.2 Å along the 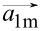 and 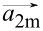 directions, respectively. In our images, although the two ends of the molecule are chemically different, they appear with equal contrast.

In order to completely understand the self-organization of MSPS on KCl there are two additional points that must be clarified: First, is the observed Moiré pattern an effect of coincidences between the quadratic lattices of molecules and substrates, and can the molecular lattice be regarded as being incompressible (i.e., the intermolecular interactions are much stronger than the molecule–substrate interactions)? Second, is the orientation of the molecular layers along the 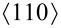 directions of the substrate due to a registry between the lattices of the substrate and the molecule (i.e., the gain in adsorption energy for a *point-on-line* coincidence as described in [9,19]), or is it due to the electrostatic interaction between the molecular dipole and the rows of charges present along the 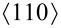 direction?

The first question of whether the Moiré pattern is formed due to coincidences between the two quadratic lattices of the substrate *c*_KCl_ and the molecules *c*_msps,exp_ can be easily answered as follows: The MSPS lattice is overlaid on the lattice of KCl with parallel orientation as observed in the experiment. Figure 3d shows a schematic representation (SPlot by Stefan C. B. Mannsfeld, Stanford Synchrotron Radiation Lightsource, Menlo Park, CA, USA) in which each circle corresponds to a protrusion in the MSPS lattice. Its color is varied as a function of the distance between its center and the position of the underlaying substrate ion. The darker the spot, the better the coincidence between the adsorbed molecule and the underlying substrate ion. Only one type of substrate ion is considered, as the electric charge of the N^+^ close to the surface (see Figure 1a) will most probably adsorb on an anion Cl^−^ and not on a cation K^+^. Figure 3d clearly shows that the experimental Moiré pattern is perfectly reproduced and, thus, the observed pattern can be explained by a simple coincidence between two parallel quadratic lattices. Note that the closer the two lattice parameters of MSPS and the substrate are, the larger the scale of the Moiré pattern will be. In order to verify if the organic layer is incompressible, we deposited and annealed sub-ML of MSPS on the NaCl, RbCl, and KBr substrates, which present significantly different lattice constants compared to KCl (Table 1). As can be seen from the values of the measured MSPS lattice constant *c*_msps,exp_ in Table 1, for the substrates KCl, RbCl, and KBr, all measured *c*_msps,exp_ are within ±1% error of 5.15 Å. The fact that no large-scale ordering is found for NaCl will be discussed below.

**Table 1:** Experimental and calculated MSPS lattice constants *c*_msps_ and Moiré pattern distances *l*_Moiré_. For the calculated values a 2-D coincidence is assumed for (*n* − *x*) MSPS/*n* ionic distances; *x* = 1 or 2.

substrate	NaCl	KCl	RbCl	KBr

*c*_sub_	3.98 Å	4.44 Å	4.62 Å	4.67 Å
*c*_msps,exp_	—	5.13 ± 0.1 Å	5.2 ± 0.1 Å	5.2 ± 0.3 Å
*l*_Moiré,exp_	—	30 ± 3 Å	38 ± 4 Å	50 ± 5 Å
*c*_msps_/*c*_sub_	11/9	7/6	9/8	11/10
*c*_msps,calc_	5.12 Å	5.18 Å	5.2 Å	5.14 Å
*l*_Moiré,calc_	44 Å	31 Å	42 Å	51 Å

In order to answer the second question of whether the orientation of the organic overlayer is determined by a pure topographic effect of the quadratic substrate lattice or if it is the lines of equal charges on the ionic substrate orient along the 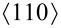 direction, we have to further evaluate the adsorption of MSPS on the substrates of KCl, RbCl, and KBr. If it is not the electrostatic MS interaction but a pure geometric effect that dominates the adsorption of MSPS on ionic substrates, one would expect that the orientation of the rectangular MSPS islands should vary for the different substrates (see Introduction and [9,19]).

Figure 3e,f and Figure 3g,h show large-scale and molecular-scale topography images for MSPS adsorbed and annealed on RbCl and KBr surfaces, respectively. On both substrates, the alignment of the molecular islands is parallel to the 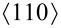 direction of the substrate, which clearly indicates that it is the electrostatic MS interaction between a molecular charge distribution and the substrate surface that dominates the self-organization of these molecules. A detailed evaluation of the observed lattice constants *c*_msps,exp_ for the different substrates as well as the Moiré pattern *l*_Moiré,exp_ is depicted in Table 1. It should be remembered that the rectangular molecular unit cell 
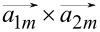
 measures two *c*_msps,exp_ distances along 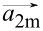 and one along 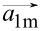 (see Figure 4).

A comparison of the experimentally determined values *c*_msps,exp_ and *l*_Moiré,exp_ with the ones calculated assuming that there is an exact coincidence for (*n* − 1) molecular protrusions with *n* substrate ions, shows that the calculated *c*_msps,calc_ maintains an almost constant value within 5.15 Å ± 1% and that the calculated parameters of the Moiré pattern are in excellent agreement with the experimental values for the substrates of KCl, RbCl, and KBr, respectively (see Table 1).

For NaCl, however, there is no coincidence for (*n* − 1) distances *c*_msps_ with *n* substrate distances, which would result in a lattice constant *c*_msps_ of close to 5.15 Å (rigid monolayer of MSPS as observed on the other substrates), but only for (*n* − 2) molecular protrusions (11 interionic distances for 9 molecular distances would give *c*_msps,calc_ = 5.12 Å; see Table 1). In order to illustrate why an *n* − 1 coincidence, but not an *n* − 2 coincidence, would show large-scale ordering, we used the following one-dimensional model for the cases of KBr (i.e., 10 *c*_msps_ for 11 substrate distances) and NaCl (i.e., 9 *c*_msps_ for 11 substrate distances) along both molecular axis directions as depicted in Figure 4.

**Figure 5:**
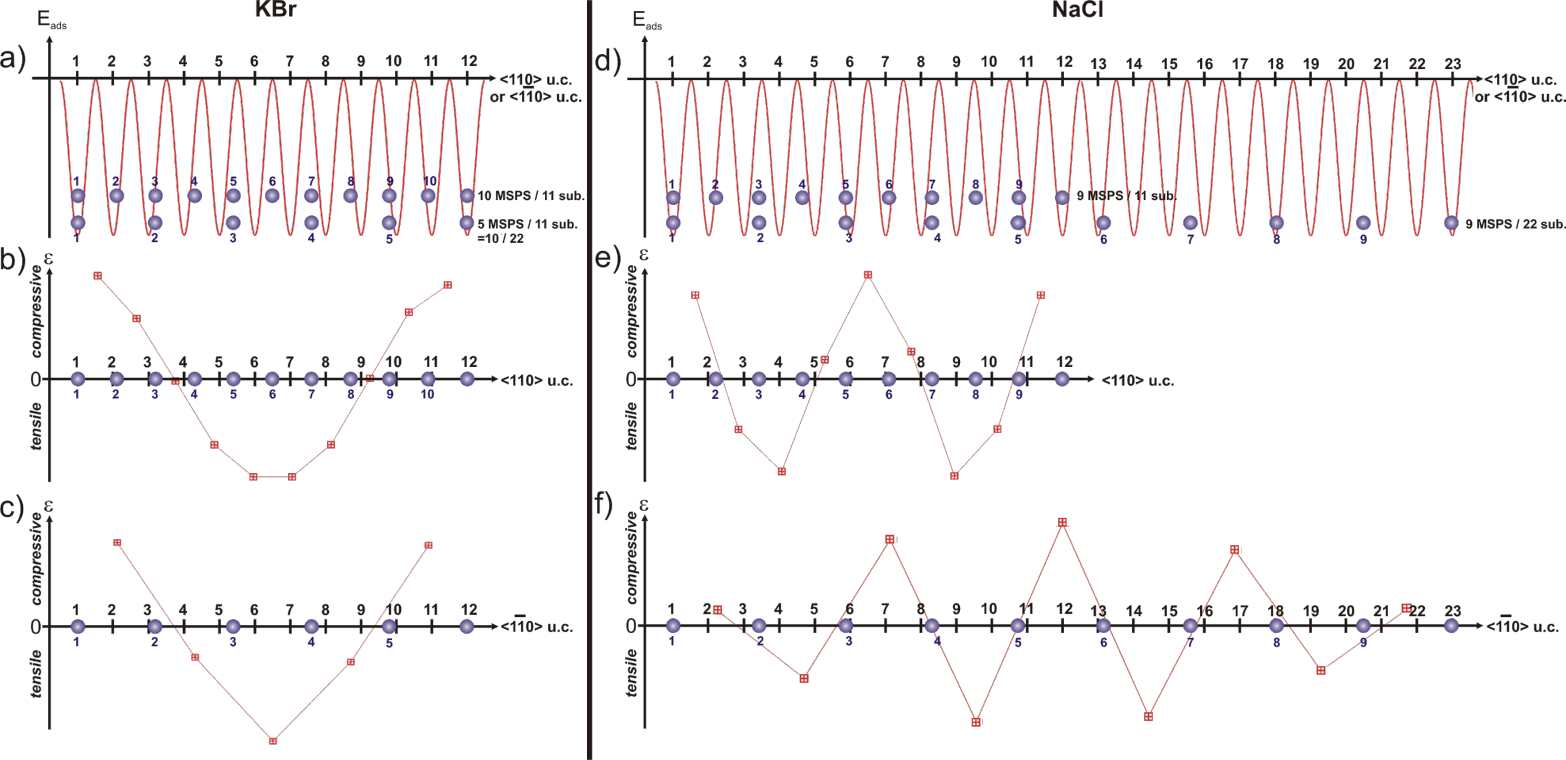
Model for the lateral stress ε in a MSPS film adsorbed on KBr (left) and NaCl (right). (a) and (d) display the position of the adsorbed molecule with respect to the sinusoidally shaped *E*_ads_ potential; (b) and (e) indicate if there is tensile or compressive stress within the film along the short molecular axes *a*_1_*_m_* (i.e., the 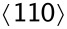 direction), and (c) and (f) along the long molecular axes *a*_2_*_m_* (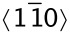 direction).

First, we assume that the adsorption energy *E*_ads_ varies laterally following the Madelung surface potential of the substrate, i.e., a sinusoidal potential, and we position the molecules according to the two coincidences (Figure 5a and Figure 5d). Along the short molecular axes, each molecular protrusion corresponds to an anchoring site (i.e., the dipolar end with the pyridine ring and its positive charge N^+^ adsorbed on an anion Cl^−^). The case for the long molecular axes, in which only every second molecular protrusion corresponds to an anchoring point, will be discussed below. Second, we calculate the lateral force that would act on a molecule within that potential (i.e., the derivative −∂*E*_ads_/∂*x*) for each discrete molecular position. Finally, we plot the difference of these discrete forces between two neighboring molecules in order to get an estimate for the local stress ε within that intermolecular bond. This stress competes with the stabilizing MM interaction. Although the film is incompressible, we assume that it can be pulled apart by tensile stress under certain circumstances as explained below.

For KBr we observe one area of tensile stress, which is centered between two areas of compressive stress. The latter are anchored on the substrate, as the first and last molecule of the row are above their preferred adsorption sites, at which the positive charge of the zwitterion would be strongly adsorbed on an anion Cl^−^ (Figure 5a and Figure 5b). For NaCl there is a double modulation of the stress ε within the film along the 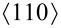 direction (Figure 5e). In contrast to the case of KBr described above, these areas of tensile stress are only anchored on one side. The area of compressive stress in the middle is not anchored to the substrate as the molecules are not above their preferred adsorption site but only close to it. Therefore, for MSPS adsorbed on NaCl, the intermolecular bonds will be locally ruptured and the long-range ordering will be perturbed, and thus no Moiré pattern will be visible. All the same, we assume that the strong electrostatic MS interaction will still force small domains or lines of molecules to arrange along the Cl^−^ lines upon annealing; however, there are too many dislocations, such that only small areas with a more or less uniform height can be observed (not shown).

Along the long molecular axes (i.e., the 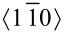 direction), only every second molecular protrusion could act as an anchoring point. As depicted in Figure 5c for KBr, this does not change the shape, with a single modulation of the stress compared to the short molecular axes for the substrates with an even number of molecular protrusions per *l*_Moiré_. However for the NaCl substrate with an odd number of protrusions per *l*_Moiré_, the N^+^ charge would be on site only after two distances *l*_Moiré_, and thus, the stress along this direction shows a very inhomogeneous modulation as depicted in Figure 5f.

The experimental results on the four ionic crystal surfaces described above clearly indicate that it is the coincidence between lines of dense molecular rows and the 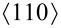 direction of the substrate that dominates the adsorption of the zwitterionic MSPS on ionic-crystal surfaces. The fact that the 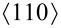 direction of the substrate presents lines of equally charged ions underlines the fact that it is the electrostatic MS interaction that determines the self-organization of MSPS, with its electric dipole moment perpendicular to the substrate surface. We therefore conclude that the observed overlayer is a *coincidence II* epitaxy, when we follow the classification scheme by Hooks et al. [2]. The characteristics of such a type of epitaxy are that only some of the overlayer lattice points lie on primitive substrate lattice lines and that a supercell can be constructed. The condition for a supercell in our case requires that the reciprocal-space lattice vectors for the substrate a^*^ and the molecular layer b^*^ satisfy the following criterion b^*^ = *f*a^*^ , with *f* being a fractional number (see the Fourier transform in Figure 3c). Finally, the fact that the molecular lattice constant almost does not change on the different investigated surfaces is clear proof that the molecular layer is incompressible and that the lateral molecule–molecule (MM) interaction is quite strong.

**Figure 6:**
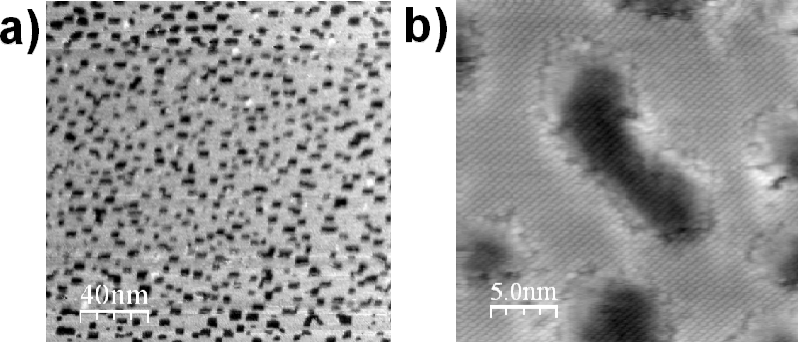
(a) Large-scale topography image of electron-bombarded KCl showing the characteristic holes (Δ*f* = −12 Hz, *A*_0_ = 5 nm); (b) topography image after deposition of ≈0.1 ML MSPS and subsequent annealing (Δ*f* = −24 Hz, *A*_0_ = 3 nm). 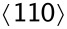 oriented step edges are visible, which are decorated with a few molecules (black dots).

As mentioned above, we observe a rearrangement of the substrate surface during the annealing cycle. The observed effect is especially significant for surfaces with low MSPS coverage. It is most likely that, during the annealing cycle, as the molecules prefer to adsorb at step edges and as the steps are not completely decorated, the molecules diffuse along the steps and do not self-organize but, rather, modify the sample topography. In order to observe in more detail how the molecules rearrange the step edges, we prepared a KCl surface with quadratic holes (produced by electron-beam irradiation, see references in [5]) shown in Figure 6a. After deposition of ≈0.1 ML of MSPS and subsequent annealing, the substrate exhibited holes in KCl with many of the step edges oriented along 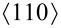 directions and which were most probably decorated with MSPS molecules (visible as black dots, Figure 6b). 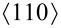 oriented steps are polar and thus energetically unfavorable; we therefore assume that the originally 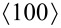 and 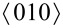 oriented step edges change their orientation to the 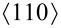 and 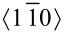 direction in order to compensate for the strong electric dipole moment of the adsorbed MSPS. A similar rearrangement of an ionic surface was observed for truxenes on KBr by Trevethan et al. [25]. In these experiments, the restructuring of rectangular edges to round structures is attributed to the fact that these molecules interact more strongly with kink and inner corner sites of a certain polarity.

## Conclusion

In summary, we have shown that zwitterionic MSPS adsorbs most probably in an isomerized agraffe-like conformation on ionic-crystal surfaces, with its electric dipole moment perpendicular to the substrate surface. We observe homogeneous and incompressible monolayers of MSPS on KCl, RbCl, and KBr substrates. Our experiments clearly indicate that it is the electrostatic molecule–substrate (MS) interaction between the positive charge of the zwitterion and the negatively charged anion of the substrate surface that determines the adsorption of MSPS in a large-scale quadratic supercell (type II coincidence [2]). For all three substrates, dense molecular rows follow the 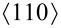 and 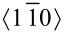 directions of the substrate, with every sixth (KCl) to tenth (KBr) molecule in coincidence with a corresponding substrate ion. It is this coincidence together with the large-scale organization that creates the experimentally observed Moiré pattern. Although, the electrostatic MS interaction dominates the adsorption mechanism, a large-scale organization of MSPS can only take place if a reasonable coincidence is possible along the 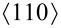 direction of the substrate, which was not the case for NaCl substrates on which the inhomogeneous stress within the molecular layer made a large-scale organization impossible. Finally, the strong molecular dipole moment can interact with the substrate in such a way that during annealing, molecules diffuse along substrate step edges and induce a reorientation of the steps in order to compensate for the electrostatic field of the adsorbed molecular dipole.
